# Lentil Waste Extracts for Inflammatory Bowel Disease (IBD) Symptoms Control: Anti-Inflammatory and Spasmolytic Effects

**DOI:** 10.3390/nu16193327

**Published:** 2024-09-30

**Authors:** Maria Antonietta Panaro, Roberta Budriesi, Rosa Calvello, Antonia Cianciulli, Laura Beatrice Mattioli, Ivan Corazza, Natalie Paola Rotondo, Chiara Porro, Antonella Lamonaca, Valeria Ferraro, Marilena Muraglia, Filomena Corbo, Maria Lisa Clodoveo, Linda Monaci, Maria Maddalena Cavalluzzi, Giovanni Lentini

**Affiliations:** 1 Department of Biosciences, Biotechnologies and Environment, University of Bari, 70125 Bari, Italy; mariaantonietta.panaro@uniba.it (M.A.P.); rosa.calvello@uniba.it (R.C.);; 2 Department of Pharmacy and Biotechnology, Food Chemistry and Nutraceutical Lab, Alma Mater Studiorum—University of Bologna, 40126 Bologna, Italy; roberta.budriesi@unibo.it (R.B.); laura.mattioli13@unibo.it (L.B.M.); 3 Department of Medical and Surgical Sciences (DIMEC), Alma Mater Studiorum—University of Bologna, 40138 Bologna, Italy; ivan.corazza@unibo.it; 4 Department of Pharmacy—Drug Sciences, University Aldo Moro-Bari, 70126 Bari, Italy; natalie.rotondo@uniba.it (N.P.R.); valeria.ferraro@uniba.it (V.F.); marilena.muraglia@uniba.it (M.M.); filomena.corbo@uniba.it (F.C.); giovanni.lentini@uniba.it (G.L.); 5 Department of Clinical and Experimental Medicine, University of Foggia, 71100 Foggia, Italy; chiara.porro@unifg.it; 6 Institute of Sciences of Food Production, National Research Council of Italy (CNR-ISPA), 70126 Bari, Italy; antonella.lamonaca@ispa.cnr.it (A.L.); linda.monaci@ispa.cnr.it (L.M.); 7 Department of Soil, Plant and Food Sciences, University Aldo Moro-Bari, 70126 Bari, Italy; 8 Interdisciplinary Department of Medicine, University of Bari, 70124 Bari, Italy; marialisa.clodoveo@uniba.it

**Keywords:** lentil hulls, inflammatory bowel diseases (IBDs), microwave-assisted extraction (MAE), circular economy, cytokines, intestinal epithelial cells, ileum and colon contractility, liquid chromatography-high resolution mass spectrometry (LC-HRMS)

## Abstract

Background/Objectives: In the contest of agro-industrial waste valorization, we focused our attention on lentil seed coats as a source of health-promoting phytochemicals possibly useful in managing inflammatory bowel diseases (IBDs), usually characterized by inflammation and altered intestinal motility. Methods: Both traditional (maceration) and innovative microwave-assisted extractions were performed using green solvents, and the anti-inflammatory and spasmolytic activities of the so-obtained extracts were determined through in vitro and ex vivo assays, respectively. Results: The extract obtained through the microwave-assisted procedure using ethyl acetate as the extraction solvent (BEVa) proved to be the most useful in inflammation and intestinal motility management. In LPS-activated Caco-2 cells, BEVa down-regulated TLR4 expression, reduced iNOS expression and the pro-inflammatory cytokine IL-1 production, and upregulated the anti-inflammatory cytokine IL-10 production, thus positively affecting cell inflammatory responses. Moreover, a significant decrease in the longitudinal and circular tones of the guinea pig ileum, with a reduction of transit speed and pain at the ileum level, together with reduced transit speed, pain, and muscular tone at the colon level, was observed with BEVa. HPLC separation combined with an Orbitrap-based high-resolution mass spectrometry (HRMS) technique indicated that 7% of all the identified metabolites were endowed with proven anti-inflammatory and antispasmodic activities, among which niacinamide, apocynin, and *p*-coumaric acid were the most abundant. Conclusions: Our results suggest that lentil hull extract consumption could contribute to overall intestinal health maintenance, with BEVa possibly representing a dietary supplementation and a promising approach to treating intestinal barrier dysfunction.

## 1. Introduction

Inflammatory bowel diseases (IBDs) are a group of multifactorial immune-mediated disorders causing chronic inflammation of the gastrointestinal tract (GI). They are defined as inflammatory syndromes since they are characterized by sustained inflammation due to the activation of the immune system by agents that are not yet well-identified, and chronic because they are long-course diseases with the likelihood of recurrent symptoms at any time in the patient’s life [1]. IBD incidence is rising worldwide, with a high burden in North America and Western Europe [2]. Crohn’s disease (CD) and ulcerative colitis (UC) represent the two main subtypes [3]. In general, the main symptoms include diarrhea, abdominal pain, blood in the stool, weight loss, and fatigue, with complications ranging from intestinal stenosis to an increased risk of colorectal cancer [4]. The activation of the innate immune system leads to excessive production of proinflammatory cytokines and mediators (such as IL-1 and nitric oxide), resulting in recruiting more immune cells into the mucosa and perpetuating intestinal inflammation [5]. The study of the inflammatory mechanisms allows for the identification of targets for therapies capable of selectively inhibiting the main signaling pathways of the inflammatory process. Although several therapies are available to reduce inflammation, including corticosteroids, 5-aminosalicylates, immunomodulators, biologic drugs, and the recently developed Janus Kinase (JAK) inhibitors [6], serious side effects are often associated with their long-term use. Ongoing advances are, therefore, needed in IBD treatment, and attention is also being paid to non-pharmacological adjuvant therapies that play a pivotal role in relieving symptoms and maintaining general well-being. Being the diet significantly involved in the etiology and symptoms of IBDs, although association but not causality is often pointed out, the role of diet and nutrition is gaining growing interest in the modulation of intestinal inflammation [7,8]. Some functional foods and crude extracts have been recently investigated for their potential effects in managing intestinal inflammation [9] and IBD symptoms [10,11,12,13,14,15]. On the other hand, several classes of isolated natural compounds, including alkaloids, polyphenols, flavonoids, terpenoids, glycosides, and bioactive peptides, have demonstrated in vivo therapeutic activity against experimental IBD models through multiple pathways [16]. Although lentils (*Lens culinaris*; Family: Fabaceae) are recognized as a functional food due to their health benefits and nutritional properties, studies on the beneficial effects of their extracts on IBD-related anti-inflammatory properties are still limited and deserve a deeper investigation [17,18,19,20,21]. Since agro-industrial waste valorization is on the cutting edge of green innovation, offering a viable solution for waste reduction and resource optimization by converting agricultural and industrial by-products into valuable resources [22,23,24,25], we focused our attention on lentil seed coats, the outer layers removed during the decortication process of lentils and often discarded as waste, to evaluate their potential as a source of health-promoting phytochemicals possibly useful in managing IBDs. Based on the knowledge that diarrhea, abdominal cramps, and pain are the most common IBD symptoms [26], this study aimed to evaluate not only the anti-inflammatory effect of different lentil hull extracts but also a possible spasmolytic effect on intestinal smooth muscle, the latter having never been reported so far. In particular, we studied the lentil hull extracts’ effect on the inflammatory responses using a human colorectal cell line (Caco-2) stimulated with lipopolysaccharide (LPS) to mimic conditions of infection and inflammation; the effect on the modulation of both spontaneous and induced intestinal contractility, which play a pivotal role in the control of IBDs, was also investigated. Metabolomic characterization of the most potent extract was performed by combining HPLC separation with an Orbitrap-based high-resolution mass spectrometry (HRMS) technique.

## 2. Materials and Methods

### 2.1. Lentil Materials

Lentils (*Lens culinaris* var. Eston green) were harvested in 2021 from Piana Cardone in Agro di Genzano (Lucania cultivar, Basilicata, Italy) and were kindly provided by Agricola Piana d’oro. In particular, lentil seed coats were separated from the cotyledons by using a semi-industrial husker from Baragioli mechanical workshop of Baragioli Marco & c. s.n.c. (Vercelli, Italy) at a rotor speed of 7.5 KW power. The obtained hull was collected, vacuum-packed in plastic bags, and stored at room temperature until use.

### 2.2. Lentil Hull Extraction Procedures

#### 2.2.1. Conventional Extraction

A suspension of 200 g of hull powder in 1 L of the appropriate solvent (abs EtOH or EtOAc; see Table 1) was stirred at room temperature for 24 h. The solution was then filtered through a Whatman (No. 1) filter paper and centrifuged at 8000 rpm (10 min, 25 °C), and the solvent was evaporated under reduced pressure using a Büchi rotavapor R-124 equipped with a Büchi waterbath B-480 and a Büchi vacuum pump V-100 (BüchiItalia s.r.l., Cornaredo, Italy); the so-obtained samples were stored at −20 °C until analysis.

#### 2.2.2. Microwave-Assisted Extraction (MAE)

A closed-system MAE was carried out at a constant temperature under continuous stirring in a CEM Discover Bench Mate microwave reactor equipped with Synergy software 1.39. The temperature was measured and controlled using a built-in infrared detector. Briefly, 400 mg of lentil hull powder in 2 mL of the appropriate solvent (abs EtOH or EtOAc; see Table 1) was irradiated using microwaves at 80 °C for 5 min. The solution was then filtered through a Whatman (No. 1) filter paper and centrifuged at 8000 rpm (10 min, 25 °C), and the solvent was evaporated under reduced pressure. The samples were stored at −20 °C until needed for analysis.

### 2.3. Determination of Total Phenolic Content (TPC)

The total phenolic content (TPC) of the lentil hull extracts was determined as previously described [27]. More specifically, the assessment of Folin Ciocalteu (F–C) reduction capacity was performed on a microplate reader (Tecan Infinite Pro 200 Microplate Reader) using spectrophotometric detection and 96-well microtiter plates (Greiner 96 Flat Bottom Transparent Polystyrene, Sigma-Aldrich, St. Louis, MO, USA). Hence, 12.5 µL of a standard gallic acid (GA) solution or diluted lentil extracts were placed in each well. Subsequently, 12.5 µL of MeOH in 50 µL of distilled water, 12.5 µL of F–C reagent, and 112.5 µL of distilled water were added to each well. After 5 min, 50 µL of a Na_2_CO_3_ solution (20%) was added to each well, and the mixture was incubated for 90 min at 30 °C. Then, the absorbance values were registered at λ = 700 nm. A series of GA standard solutions at different concentrations (0.025–0.25 mg/mL) was prepared and used to construct a calibration curve (y = 3.3467x + 0.0018, R^2^ = 0.9814). MeOH was used as a blank. The TPC value was expressed as gallic acid equivalent (GAE) milligrams per g of lentil extract (mg GAE/g).

### 2.4. Anti-Inflammatory Activity

#### 2.4.1. Cell Cultures and Treatments

Caco-2 human colorectal cancer cells (ICLC HTL 97023), representing a well-established intestinal-like in vitro model [28], were obtained from Interlab Cell Line Collection (Genoa, Italy) and were cultured in a MEM medium supplemented with 10% fetal bovine serum (FBS, UE approved origin), 100 U/mL penicillin, 100 μg/mL of streptomycin, l-glutamine (2 mM), and 1% nonessential amino acids (NEEAs), (all reagents were purchased from Life Technologies-Invitrogen, Milan, Italy), referred to as the complete medium.

Cell cultures were maintained at 37 °C in a humidified atmosphere containing 5% CO_2_ and expanded in tissue culture flasks (75 cm^2^, BD Biosciences, Milan, Italy), refreshing the medium once a day. The cells were seeded in six-well cell culture plates and 96-multiwell plates, cultured to reach 80% confluency, and then submitted to subsequent treatments.

For the experiments, cells were treated with 1 μg/mL *Salmonella enterica typhimurium* LPS (Sigma-Aldrich, Milan, Italy) for 24 h according to preliminary experiments. Before LPS stimulation, some wells were pre-treated with different concentrations (100, 10, 1, 0.25 μg/mL) of each extract and reference compounds (BEVb, BEVa, mBEVb, mBEVa; see Table 1). After 1 h of incubation at 37 °C, cell cultures were stimulated with LPS as previously indicated. Untreated cells were used as control.

#### 2.4.2. Cell Viability Assay

To test cell viability, we used the (3,4,5-dimethylthiazol-2-yl)-2-5-diphenyltetrazolium bromide (MTT) assay based on the reduction of MTT by the mitochondrial dehydrogenase to a purple formazan product in vital cells (Sigma-Aldrich) [29]. Briefly, cells (1 × 10^4^) were seeded in a 96-well plate (BD Biosciences). After cell treatment, culture media were carefully removed, and 100 μL of 0.5 mg/mL MTT in cell culture medium was added to each well. At the end of the incubation of 4 h, 100 μL of a solution of 10% SDS, 0.01 M HCl, was added to each well to dissolve the formed formazan crystals. Cell viability was measured by reading the absorbance (560 nm) on Cytation 3 Cell Imaging Multi-Mode Reader (Biotek, Winooski, VT, USA). The cell viability was calculated according to the following formula:

$$\%\;cell\;viability=\frac{OD\;\left(560\;nm\right)\;tested\;compound}{OD\;\left(560\;nm\right)\;control\;cells}\times 100$$

Values were expressed as the average percentage ± SD, where OD represents the optical density indicated between parentheses.

#### 2.4.3. Electrophoresis and Western Blotting

After in vitro treatments, cells were lysed for 30 min on ice with lysis buffer [1% Triton X-100, 20 mM Tris–HCl, 137 mM NaCl, 10% glycerol, 2 mM EDTA, 1 mM phenylmethylsulfonyl fluoride (PMSF), 20 μM leupeptin hemisulfate salt, 0.2 U/mL aprotinin (Sigma-Aldrich, St. Louis, MO, USA)]. The lysate, vortexed for 15–20 s, was centrifuged at 12,800× *g* for 20 min. The protein concentration in the supernatant was spectrophotometrically determined with Bradford’s protein assay. Briefly, protein samples were diluted with a sample buffer (0.5 M Tris HCl pH 6.8, 10% glycerol, 10% *w*/*v* SDS, 5% 2-mercaptoethanol, 0.05% *w*/*v* bromophenol blue) and then boiled for 3 min. Proteins (25 μg/lane) and pre-stained standards (BioRad Laboratories, Hercules, CA, USA) were loaded on 4–12% SDS precast polyacrylamide gels (Life Technologies, Milan, Italy).

The electrophoretic bands were transferred on a nitrocellulose membrane, blocked with 5% (*w*/*v*) non-fat dried milk for 1 h, and then washed 3 times with 0.1% Tween 20-PBS (T-PBS). Then, following a standard avidin-biotin complex procedure, membranes were incubated in the dark with primary mouse monoclonal antibody (moAb) anti-β-actin (sc-47778), rabbit poAb anti-TLR4 (sc-10741) (all from Santa Cruz Biotechnology, Inc., Milan, Italy), at a 1:500 dilution. The membranes were washed with T-PBS (for 20 min, 3 times) and then incubated for 60 min at room temperature in the dark on a shaker with the secondary antibody anti-mouse or anti-rabbit IgG, horseradish peroxidase (HRP)-conjugate (Bentham, Milan, Italy) diluted 1:10,000. After three washes with 0.1% T-PBS, immunoreactive bands were acquired by ChemiDoc XRS+ Imager (Bio-Rad Laboratories, Inc., Hercules, CA, USA), and we quantified the optical density of bands normalized with β-actin to be expressed as mean ± SD. Bands were visualized with the chemiluminescence method (BioRad Laboratories).

#### 2.4.4. Reverse Transcriptase-Polymerase Chain Reaction (RT-PCR) and Quantitative Real-Time PCR Analyses

Total cell RNA was extracted by using GenElute™ Mammalian Total RNA Miniprep Kit (Sigma-Aldrich) according to the manufacturer’s instructions. Then, RNA was reverse-transcribed back into cDNA with SuperScript III Reverse Transcriptase (Thermo Fisher Scientific, Milan, Italy), and the expression rates of the mRNA levels of various genes were quantified using the SYBR Green QuantiTect RTPCR Kit (Roche, San Francisco, CA, USA). β-actin was used as an endogenous reference. Data were analyzed using the relative standard curve method according to the manufacturer’s protocol. The mean value of each gene after β-actin normalization at the time point showing the highest expression was used as a calibrator to determine the relative levels. The primers used for amplification were iNOS (NM_000625.4) forward primer 5′-CAGGAGGATGCCTTCCGCAGCTGG-3′, reverse primer 5′-ATGATGAGGTAGTCGAGGAGGGTC-3′; IL-1β (XM_047444175.1) forward primer 5′-CACGATGCACCTGTACGATCA-3′, reverse primer 5′-GTTGCTCCATATCCTGTCCCT-3′; IL-10 (NM_000572.3) forward primer 5′-AGAACCTGAAGACCCTCAGGC-3′, reverse primer 5′-CCACGGCCTTGCTCTTGTT-3′; and for β-actin (NM_001101.5) forward primer 5′-GGCGGCACCACCATGTACCCT-3′, reverse primer 5′-AGGGGCCGGACTCGTCATACT-3′. One microliter of cDNA was amplified in 25 μL of PCR solution (11.5 μL of cDNA solution in water, 1 μL of primer sets, and 12.5 μL of Power SYBR Green PCR Master Mix; Thermo Fisher) in a 7500 Real-time PCR System (Applied Biosystems, Monza, Italy), and fluorescence was monitored at each cycle. Cycle parameters were 95 °C for 15 min to activate Taq, followed by 40 cycles of 95 °C for 15 s, 55 °C for 1 min, and 72 °C for 1 min. Serial dilutions of cDNA from the same source as samples were used to obtain a standard curve. The individual targets for each sample were quantified by determining the cycle threshold and by comparison with the standard curve. The relative amount of the target mRNA was normalized to the level of β-actin mRNA.

#### 2.4.5. Data Presentation and Statistical Analysis

Student’s *t*-test and analysis of variance (one-way ANOVA) on the results of at least five independent biological replicates were performed. Values of *p* < 0.05 were considered statistically significant.

### 2.5. Spasmolytic Activity

Guinea pigs of either sex (200–400 g) obtained from Charles River (Calco, Como, Italy) were used. The animals were housed according to the ECC Council Directive regarding the protection of animals used for experimental and other scientific purposes (Directive 2010/63/EU of the European Parliament and of the Council) and the WMA Statement on Animal Use in Biomedical Research. All procedures followed the guidelines of the animal care and use committee of the University of Bologna (Bologna, Italy). The ethical committee authorization was reported and numbered as “Protocol 2DBFE.N.YEV” by the Comitato Etico Scientifico for Animal Research Protocols according to D.L. vo 116/92 and approved by the Ministry of Health in December 2023.

Guinea-pig ileum. As previously described [30], the terminal portion of the ileum (3–4 cm near the ileocaecal junction) was cleaned, and segments 2–3 cm long of ileum were set up under 1 g tension at 37 °C in organ baths containing Tyrode solution of the following composition (mM): NaCl, 118; KCl, 4.75; CaCl_2_, 2.54; MgSO_4_·7H_2_O, 1.20; KH_2_PO_4_·2H_2_O, 1.19; NaHCO_3_ 25; glucose 11. When BaCl_2_ was used as the agonist, MgSO_4_·7H_2_O was replaced by MgCl_2_·6H_2_O. The two segments obtained (2–3 cm) were set up under 1 g tension in the longitudinal and circular directions along the intestinal wall. Tissues were allowed to equilibrate for at least 30 min during which time the bathing solution was changed every 10 min.

Guinea-pig proximal colon. Starting approximately 1 cm distal from the caecocolonic junction, two segments of about 1 cm of the guinea pig’s proximal colon were cut. The proximal colon was cleaned by rinsing it with De Jalon solution of the following composition (mM): NaCl, 155; KCl, 5.6; CaCl_2_, 0.5; NaHCO_3_, 6.0; glucose, 2.8; and the mesenteric tissue was removed. The two segments were suspended in organ baths containing gassed warm de Jalon solution under a load of 1 g maintained at 37 °C. Tension changes in longitudinal and circular muscle length were recorded. Tissues were allowed to equilibrate for at least 30 min, during which time the bathing solution was changed every 10 min [31].

#### Spontaneous Contractility

The experimental design was previously described [30]. Briefly, for the ileum and colon, the tracing graphs of spontaneous longitudinal and circular contractions were continuously recorded with the LabChart Software 7.0 Pro (ADInstruments, Bella Vista, New South Wales, Australia). After the equilibration period (about 30 to 45 min according to each tissue), cumulative concentration curves of extracts and reference compounds (BEVb, BEVa, mBEVb, mBEVa) were constructed. At the end of each dose, the following parameters of the spontaneous contraction (SC) recording were evaluated considering a 5 min stationary period: the mean contraction amplitude (MCA), evaluated as the mean force value (g); the standard deviations of the force values over the period, as an index of the spontaneous contraction variability (SCV); and basal spontaneous motor activity (BSMA), as the percentage (%) variation of each mean force value (g) for the control period. The spontaneous contractions were investigated in the frequency domain through a standard FFT analysis and a subsequent Power Spectral Density (PSD) plot. The absolute powers of the following frequency bands of interest—low [0.0, 0.2] Hz (LF), medium [0.2, 0.6] Hz (MF), and high [0.6, 1.0] Hz (HF)—were then calculated [30]. The PSD percentage (%) variations for each band of interest to control were estimated.

All of the calculations were carried out in a post-processing phase. To avoid errors due to the presence of artifacts, the period of analysis was chosen by a skilled operator.

Comparisons between mean spontaneous contraction amplitudes (MCA) at different concentrations were performed by one-way ANOVA with Bonferroni’s correction. Differences with *p* < 0.05 were considered statistically significant.

LF, MF, and HF contractions were considered significant when they were higher or lower than 50% variations for the basal values.

### 2.6. Lentil Hull Metabolite Profiling

#### 2.6.1. Sample Preparation

Samples of lentil hull extracts (Eston green) obtained by microwave-assisted extraction with ethyl acetate were further dissolved in MeO:H_2_O 80:20 (*v*/*v*) solvent to obtain a 10 mg/mL stock solution. After shaking, the samples were further diluted with H_2_O:MeOH (95/5, *v*/*v*) in a ratio of 1:2 (final matrix concentration 5 mg/mL). Further experimental details are reported elsewhere [27].

#### 2.6.2. Untargeted High-Resolution Mass Spectrometry Analysis

Metabolic profiling of lentil hull samples was carried out by LC-MS/MS analysis on an Ultimate 3000 UHPLC system coupled to a hybrid quadrupole-Orbitrap^TM^ mass spectrometer Q-Exactive Plus (Thermo Fisher Scientific, Bremen, Germany). Chromatographic separation of metabolites was accomplished on an Acclaim^TM^ 120, C18 analytical column (3 µm, 120 Å, 2.1 × 150 mm, Thermo Fisher Scientific, Bremen). Spectra were acquired both in the positive and negative ion modes in the mass range of 70–1050 *m*/*z* by running the instrument in a FullMS/Data-dependent acquisition mode (FullMS/DD^2^). Further experimental details are described elsewhere [27].

#### 2.6.3. Metabolite Identification

Raw ultra-performance liquid chromatography and high-resolution mass spectrometry data were acquired using Xcalibur software (version 2.1, Thermo Fisher Scientific, Bremen, Germany), while Compound Discoverer software (version 3.3.1.111 SP1, Thermo Fisher Scientific, Bremen, Germany) was used for peak alignment, background subtraction, and extraction of features. Unknown compound identification and prediction of the elemental composition were accomplished by performing the Chem-Spider and mzCloud node searches. The identification of the molecules was carried out according to the criteria established by the “Metabolomics Standard Initiative” [32,33]. In particular, the compounds were identified according to two approaches to satisfy the standard criteria of “level IIa” (the identification of the molecules is based on the comparison between the HRMS/MS fragmentation patterns of the unknown molecules with those deposited in the mzCloud database) and “level III” (putatively identification using the accurate mass obtained for each compound compared to the masses deposited in the ChemSpider database). For more reliable identification, ChemSpider and mzCloud results were visually inspected by the operator, and only those compounds fulfilling some more stringent criteria internally defined were taken into consideration. After identification, each molecule was searched in the PubChem database to retrieve the class to which the compound belongs or to obtain any other useful information to elucidate its function. Further experimental details are described elsewhere [27].

## 3. Results

### 3.1. Lentil Hull Bioactive Compounds Extraction and Determination of Total Phenolic Content (TPC)

Two different extraction procedures were carried out on Eston green lentil hulls, namely MAE and maceration. According to our previously reported procedure [27], MAE was carried out at 80 °C for 5 min and maceration at room temperature for 24 h, both procedures with a solid/liquid ratio of 200 mg/mL. As reported in Table 1, a comparable extraction yield was reached in the four extraction procedures.

The total phenolic content (TPC) of the four obtained extracts was determined by a Folin–Ciocalteu assay and, as reported in Table 1, a higher TPC was observed when abs EtOH was used in comparison with EtOAc (entries 1 and 3 compared with 2 and 4, respectively) and, in particular, when the extraction was carried out under microwave irradiation rather than maceration (entry 1 vs. 3).

### 3.2. Anti-Inflammatory Activity

#### 3.2.1. Viability Assay on the Intestinal Cells

**Figure 1:**
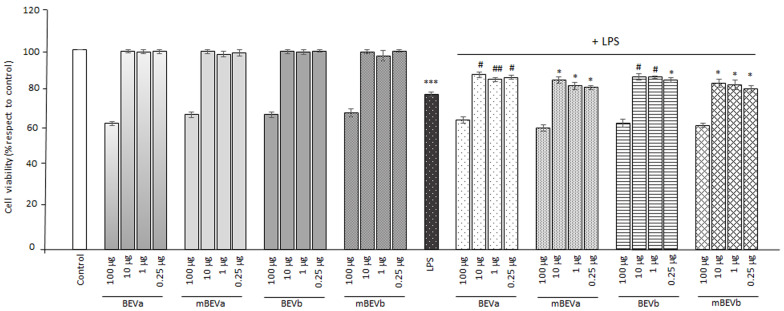
Effects of different lentil hull extracts and LPS treatment on cell viability (%). Caco-2 cells were incubated with the medium (Control), with LPS (1 μg/mL) alone, or with LPS in the presence of various lentil hull extracts at their identified optimal concentration: BEVa, mBEVa, BEVb, and mBEVb. The viable cell extent was evaluated after 24 h by the MTT assay. Values are expressed as means ± SD of five independent experiments. (*** *p* < 0.001 vs. Control; * *p* < 0.05, ^#^ *p* < 0.01, and ^##^ *p* < 0.001 vs. LPS).

Preliminary experiments on lentil hull extract cytotoxicity against Caco-2 cells were performed using the MTT assay. Cells were treated with different concentrations of BEVa, mBEVa, BEVb, and mBEVb, ranging from 100 μg/mL to 0.25 μg/mL for 24 h. Figure 1 shows that the treatment with extracts of 100 μg/mL caused significant toxicity, as observed by the reduction in the viable cells number compared to the control sample. Next, to evaluate the combined effects on cell viability, extracts were tested in the presence of LPS (1 μg/mL) (Figure 1). Caco-2 cells were pre-treated with extracts for 1 h and then induced with LPS for 24 h. In these conditions, we observed that LPS, when used alone, significantly reduced cell viability in comparison to untreated cells. Moreover, for cells submitted to the combined treatment with 100 μg/mL in the presence of LPS, we observed that the number of viable cells significantly diminished in comparison with both cells treated with LPS alone and the controls. Interestingly, we observed that all the other extract concentrations used (ranging from 10 to 0.25 μg/mL) significantly increased cell viability in comparison to cells submitted to LPS treatment alone. Due to the significant reduction of cell viability following the treatment with 100 μg/mL compared to the other treatments, the concentration of 100 μg/mL was excluded in subsequent experimental procedures.

#### 3.2.2. Expression of Toll-like Receptors 4 on the Intestinal Cells

**Figure 2:**
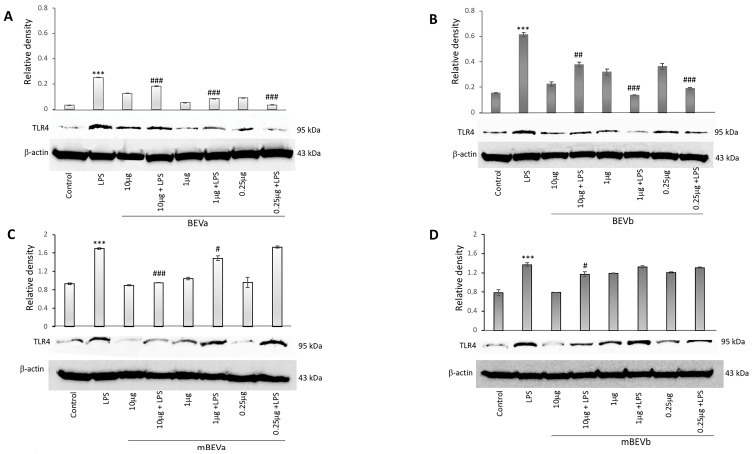
Effects of different lentil hull extracts on TLR4 expression levels in Caco-2 cells treated with LPS (1 μg/mL) for 24 h. Immunoblotting detection in the Caco-2 cells of control, LPS, BEVa and BEVa plus LPS (**A**), BEVb and BEVb plus LPS (**B**), mBEVa and mBEVa plus LPS (**C**), and mBEVb and mBEVb plus LPS (**D**). Densitometric analysis of TLR4 expression, after normalization against β-actin, is reported. Data are presented as means ± SD of five independent experiments. (*** *p* < 0.001 vs. control; ^###^ *p* < 0.001, ^##^ *p* < 0.01, and ^#^ *p* < 0.05 vs. LPS).

The regulation of TLR4 expression by lentil hull extracts was investigated by immunoblot analysis. Figure 2 shows that Caco-2 cells express this receptor, as revealed by the presence of a 95 kDa protein band corresponding to TLR4. We also observed that the expression of TLR4 in intestinal cells was significantly upregulated by LPS treatment in comparison to untreated cells. Interestingly, BEVa pre-treatment before LPS stimulation led to a significant decrease in the expression of TLR4 observed for all concentrations tested (ranging from 10 μg/mL to 0.25 μg/mL) in comparison to cells treated with LPS alone (Figure 2, panel A). The same results were determined in BEVb pre-treated Caco-2 cells stimulated with LPS. Also in this case, in fact, all concentrations tested were able to significantly reduce TLR4 expression rate in comparison to treatment of cells with LPS alone (Figure 2, panel B).

In mBEVa pre-treated cells before LPS stimulation, indeed, we observed a significant decrease in the expression of TLR4 for 10 and 1 μg/mL concentrations, whereas a 0.25 μg/mL concentration was ineffective to significantly reduce TLR4 expression (Figure 2, panel C). Finally, pre-treatment of cells with mBEVb before LPS stimulation led to a significant decrease in the expression of TLR4 only for concentrations of 10 μg/mL as reported in Figure 2, panel D. Overall, these results demonstrated that all extracts tested were able to down-regulate the TLR4 expression in LPS-stimulated cells, although not at all concentrations used. In addition, both extracts obtained under microwave irradiation (BEVa and BEVb), at all doses used, exhibited a greater ability to negatively modulate the TLR4 expression, suggesting that microwave extraction appeared to be more successful in down-regulating the expression of this endotoxin receptor.

#### 3.2.3. Effect of Lentil Hull Extracts on Inducible Nitric Oxide Synthase (iNOS) Expression in Intestinal Cells

**Figure 3:**
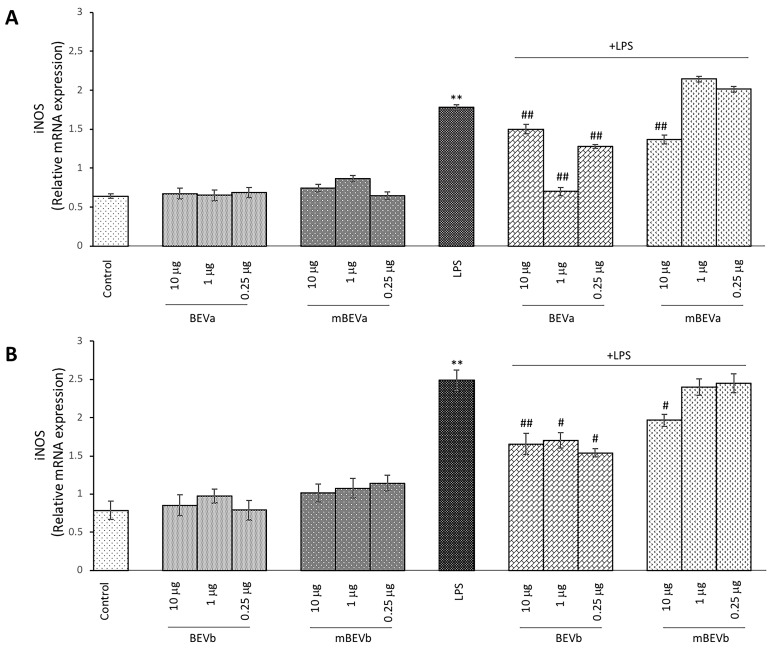
Analysis of pro-inflammatory responses. Real-time PCR analysis of iNOS mRNA expression levels in Caco2-untreated cells (control), treated with LPS alone (LPS) or treated with LPS after pre-treatment with different lentil hull extracts: (**A**) BEVa and mBEVa and (**B**) BEVb and mBEVb. Values represent the mRNA fold changes relative to β-actin used as resident control and expressed as means ± SD of five independent experiments. (** *p* < 0.05 vs. control; ^##^ *p* < 0.01 and ^#^ *p* < 0.05 vs. LPS).

To assess the modulatory effect of lentil hull extracts on the inflammatory responses of the intestinal cells activated with LPS, we also evaluated the inducible isoform of NO synthase by RT-PCR analysis. RT-PCR analysis revealed a significant increase in the iNOS mRNA in LPS-stimulated Caco-2 cells in comparison with unstimulated cells (Figure 3, panels A and B). Interestingly, BEVa pre-treatment before LPS stimulation led to a significant decrease in the expression of iNOS observed for all concentrations tested (ranging from 10 μg/mL to 0.25 μg/mL) (Figure 3, panel A). Moreover, mBEVa pre-treatment before LPS stimulation led to a significant decrease in the expression of iNOS only for 10 μg/mL, as reported in Figure 3 panel A.

In cells submitted to pre-treatment with BEVb before LPS stimulation, we observed that all concentrations tested (ranging from 10 μg/mL to 0.25 μg/mL) were able to significantly down-regulate the mRNA iNOS expression in comparison to cells treated with LPS alone (Figure 3, panel B). Finally, pre-treatment of cells with mBEVb before LPS stimulation led to a significant decrease in the mRNA iNOS expression only for concentrations of 10 μg/mL as reported in Figure 3, panel B. Overall, these data suggest that lentil hull extracts can down-regulate LPS-induced iNOS expression and that the extracts obtained under microwave irradiation revealed a more effective ability to significantly reduce this expression in comparison to the other tested extracts.

#### 3.2.4. Effect of Lentil Hull Extracts on Pro-Inflammatory IL-1 Cytokine Expression in Intestinal Cells

**Figure 4:**
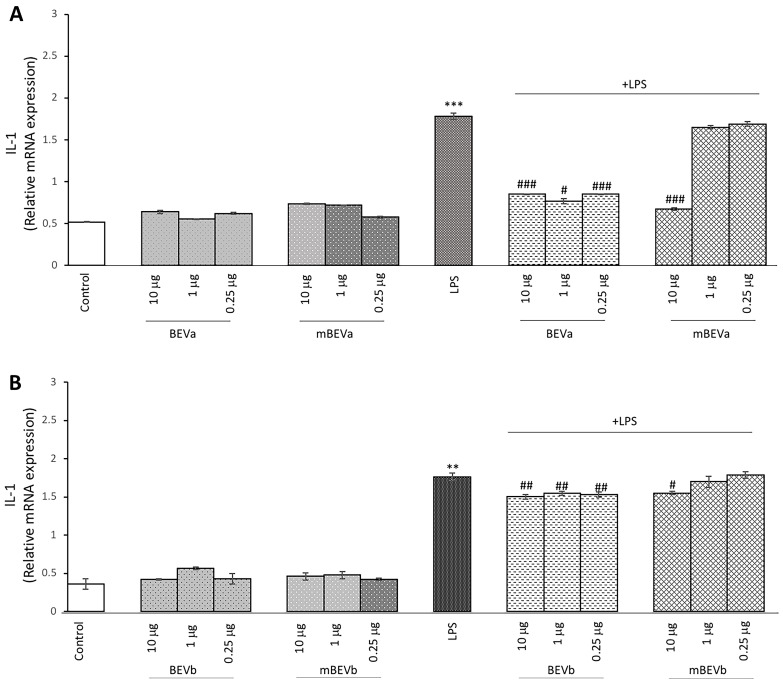
Analysis of pro-inflammatory IL-1 cytokine expression. Real-time PCR analysis of IL-1 mRNA expression levels in Caco-2 untreated cells (Control), treated with LPS alone (LPS) or treated with LPS after pre-treatment of different lentil peel extracts: (**A**) BEVa and mBEVa and (**B**) BEVb and mBEVb. Values represent the mRNA fold changes relative to β-actin used as resident control and expressed as means ± SD of five independent experiments. (*** *p* < 0.001 and ** *p* < 0.01 vs. control; ^###^ *p* < 0.001, ^##^ *p* < 0.01, and ^#^ *p* < 0.05 vs. LPS).

To further evaluate the ability to modulate cellular inflammatory response by extracts obtained from lentil hulls, their ability to modulate the expression of the pro-inflammatory cytokine IL-1 in Caco-2 cell cultures was evaluated by RT-PCR analysis. In this respect, it was shown that LPS-treated cells showed a significant increase in IL-1 expression compared to control cells (Figure 4, panels A and B). Moreover, in all treatments carried out on Caco-2 cells with the different lentil hull extracts (ranging from 10 μg/mL to 0.25 μg/mL) in the absence of LPS, we observed IL-1 levels comparable to those observed in controls (Figure 4, panels A and B). As reported in Figure 4, in LPS-stimulated Caco-2 cells, pre-treated with the different extracts, all concentrations of BEVa and BEVb were effective in significantly reducing IL-1 expression (Panels A and B). Conversely, mBEVa and mBEVb extracts only at a concentration of 10 μg/mL were able to significantly reduce IL-1 expression, compared to cells treated with LPS alone (Figure 4, panels A and B). From these data emerges that lentil hull extracts have the potential to modulate the intestinal inflammatory response. In particular, microwave-obtained extracts (BEVa and BEVb) seem to be the most effective in reducing pro-inflammatory responses in LPS-treated Caco-2 cells when compared to the extracts obtained with the conventional extraction method.

#### 3.2.5. Effect of Lentil Hull Extracts on Anti-Inflammatory IL-10 Cytokine Expression in Intestinal Cells

**Figure 5:**
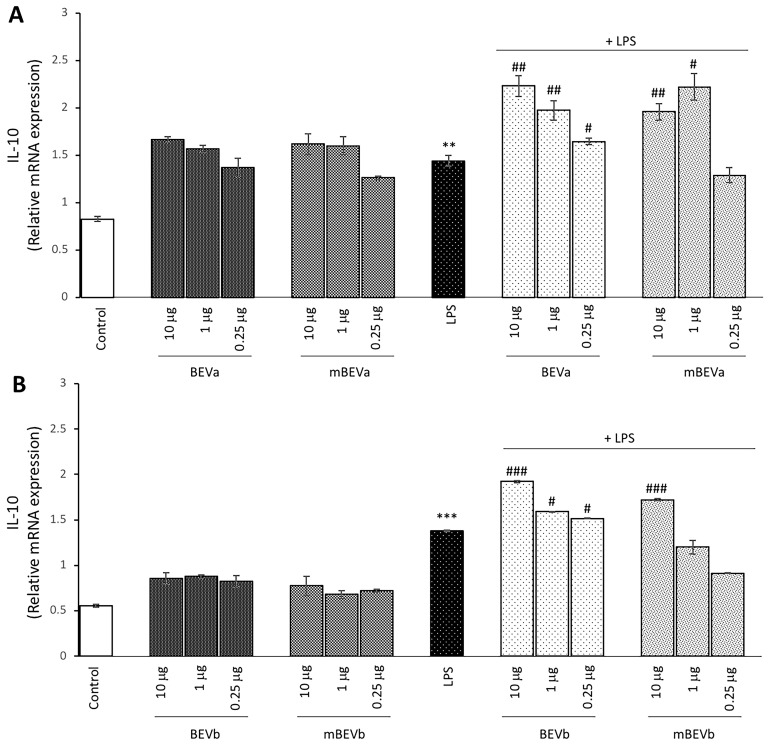
Analysis of anti-inflammatory cytokine expression. Real-time PCR analysis of IL-10 mRNA expression levels in Caco-2 untreated cells (Control), treated with LPS alone (LPS) or treated with LPS after pre-treatment of different lentil hull extracts: (**A**) BEVa and mBEVa and (**B**) BEVb and mBEVb. Values represent the mRNA fold changes relative to β-actin used as resident control and expressed as means ± SD of five independent experiments. (*** *p* < 0.001 and ** *p* < 0.01 vs. control; ^###^ *p* < 0.001, ^##^ *p* < 0.01, and ^#^ *p* < 0.05 vs. LPS).

The possible anti-inflammatory effect of lentil hull extracts on LPS-treated Caco-2 cultures was also investigated. For this purpose, the ability to modulate the expression of the anti-inflammatory cytokine IL-10 in Caco-2 cell cultures was evaluated. The analysis revealed, both for BEVa and mBEVa, as well as for BEVb and mBEVb, that all the different lentil extract concentrations used in Caco-2 cells, in the absence of LPS, were able to significantly upregulate the IL-10 expression in comparison to control cells (Figure 5, panels A and B). Moreover, the expression of IL-10 was significantly upregulated also in cells treated with LPS alone in comparison to untreated cells (Figure 5, panels A and B). Interestingly, in LPS-stimulated Caco-2 cells, pre-treated with the different extracts, all concentrations of BEVa and BEVb were able to induce a significant increase in IL-10 expression in comparison to cells treated with LPS alone. In addition, mBEVa extracts were able to significantly upregulate IL-10 expression in LPS-treated Caco-2 cells and pre-treated with 10 μg/mL and with 1 μg/mL in comparison to LPS-alone-treated cells (Figure 5, panel A). Whereas mBEVb pre-treatment before LPS stimulation determined only at 10 μg/mL concentration resulted in a significant upregulation of IL-10 expression in comparison to cells treated with LPS alone (Figure 5, panel B). Overall, lentil hull extracts seem to exhibit anti-inflammatory effects in Caco-2 cells, as demonstrated by the upregulation of the expression of the anti-inflammatory cytokine IL-10. This anti-inflammatory effect is more evident in cells submitted to a combined treatment of LPS in the presence of extracts, thus suggesting that lentil hull extracts could be useful for the containment of intestinal inflammatory responses. In addition, also in this case, the microwave-obtained extracts showed a more potent capacity to markedly upregulate the expression of the anti-inflammatory cytokine IL-10 in comparison to the extracts obtained by conventional methods.

### 3.3. Spasmolytic Activity

The four prepared lentil hull extracts were also studied for their effects on ileum and colon ex vivo contractility, focusing our attention on the spontaneous longitudinal and circular basal contractility and the potential related impacts on different physiological parameters.

#### 3.3.1. Effects of Lentil Hull Extracts on Ileum Muscles

**Figure 6:**
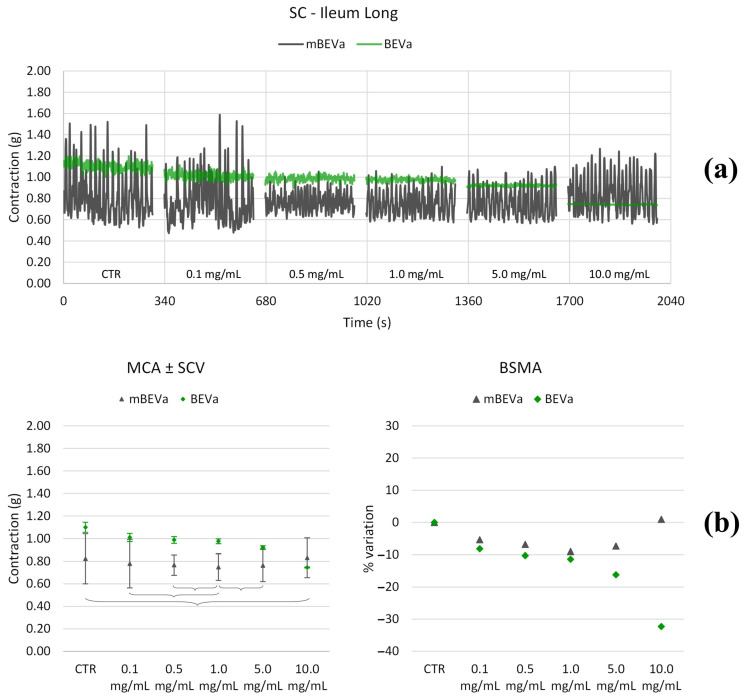
Experimental original recording of the concentration–response curve of BEVa and mBEVa on spontaneous longitudinal (Long) ileum basal contractility. (**a**) Spontaneous contraction (SC) signals for each concentration; (**b**) mean contraction amplitude (MCA) and spontaneous contraction variability (SCV), represented as error bars in the MCA plot and contraction percentage variation for the control (BSMA) for each considered condition; not significant differences (*p* > 0.05) between MCAs at different concentrations are reported in the graph. All the unreported comparisons are considered significant (*p* < 0.05).

**Figure 7:**
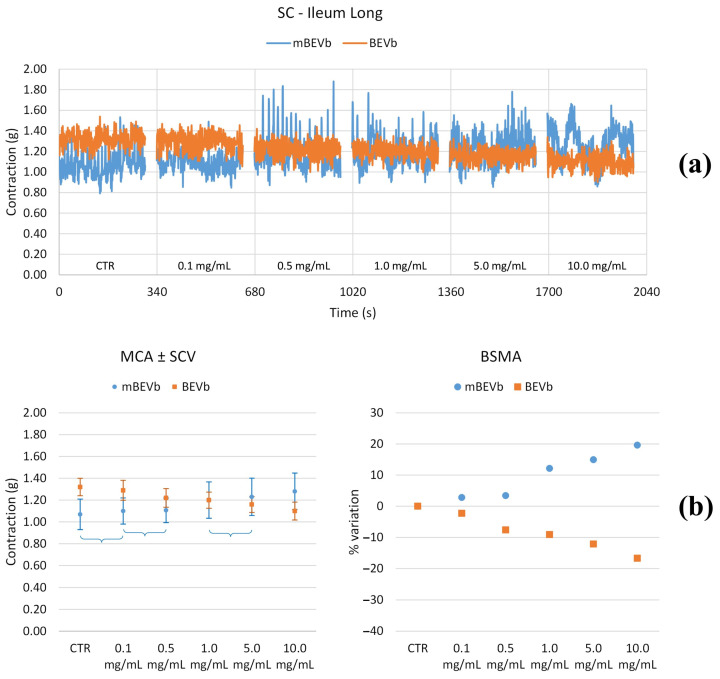
Experimental original recording of the concentration–response curve of BEVb and mBEVb on spontaneous longitudinal (Long) ileum basal contractility. (**a**) Spontaneous contraction (SC) signals for each concentration; (**b**) mean contraction amplitude (MCA) and spontaneous contraction variability (SCV), represented as error bars in the MCA plot and contraction percentage variation for the control (BSMA) for each considered condition; not significant differences (*p* > 0.05) between MCAs at different concentrations are reported in the graph. All the comparisons not reported are to be considered significant (*p* < 0.05).

**Figure 8:**
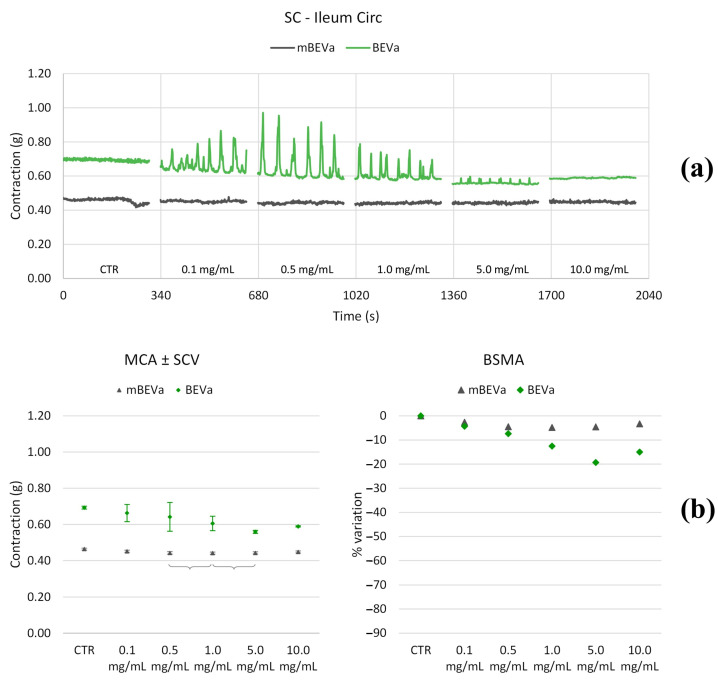
Experimental original recording of the concentration–response curve of BEVa and mBEVa on spontaneous circular (Circ) ileum basal contractility. (**a**) Spontaneous contraction (SC) signals for each concentration; (**b**) mean contraction amplitude (MCA) and spontaneous contraction variability (SCV), represented as error bars in the MCA plot and contraction percentage variation for the control (BSMA) for each considered condition; not significant differences (*p* > 0.05) between MCAs at different concentrations are reported in the graph. All the unreported comparisons are considered significant (*p* < 0.05).

**Figure 9:**
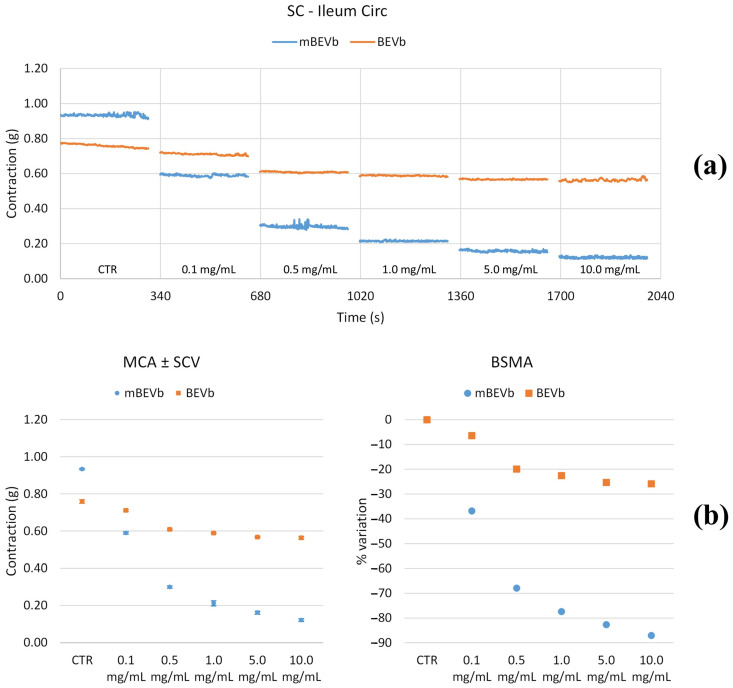
Experimental original recording of the concentration–response curve of BEVb and mBEVb on spontaneous circular (Circ) ileum basal contractility. (**a**) Spontaneous contraction (SC) signals for each concentration; (**b**) mean contraction amplitude (MCA) and spontaneous contraction variability (SCV), represented as error bars in the MCA plot and contraction percentage variation for the control (BSMA) for each considered condition; not significant differences (*p* > 0.05) between MCAs at different concentrations are reported in the graph. All the unreported comparisons are considered significant (*p* < 0.05).

As depicted in Figure 6, Figure 7, Figure 8 and Figure 9 and summarized in Table 2 and Table 3, both longitudinal and circular tones decreased significantly in the presence of BEVa, already at low concentrations; pain and transit speed decreased starting from 0.5 mg/mL and 1.0 mg/mL, respectively; mixing capacity increased at low concentrations; fragmentation decreased at 5.0 mg/mL. The corresponding macerate (mBEVa) reduced both tones, already at low concentrations, transit speed, and pain; on the contrary, mixing slightly increased. Both tones decreased in the presence of BEVb; transit speed, pain, mixing, and fragmentation showed no significant differences, except for high concentrations. Finally, as regards mBEVb, the longitudinal tone increased at low concentrations, while the circular tone decreased significantly at all concentrations; transit speed remained almost unchanged; pain and bolus fragmentation slightly increased; mixing capacity decreased.

#### 3.3.2. Effects of Lentil Hull Extracts on Colon Muscles

**Figure 10:**
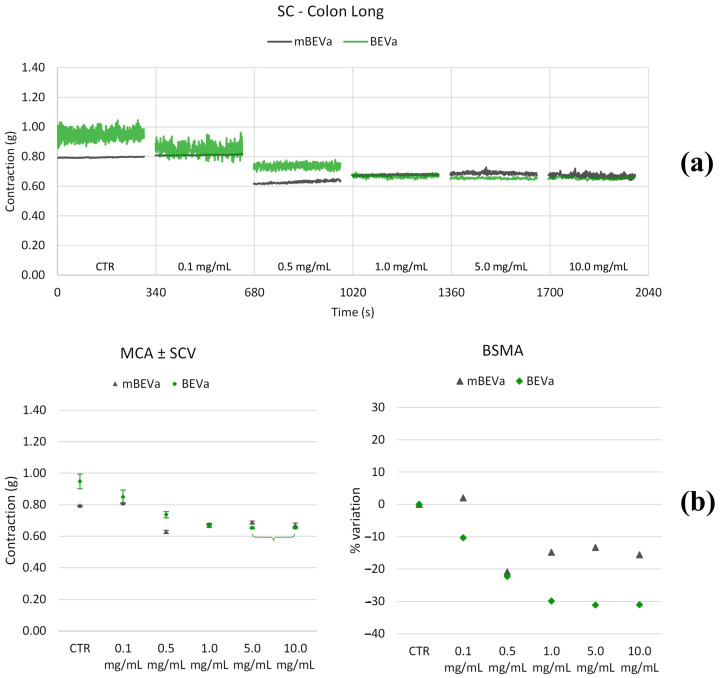
Experimental original recording of the concentration–response curve of BEVa and mBEVa on spontaneous longitudinal (Long) colon basal contractility. (**a**) Spontaneous contraction (SC) signals for each concentration; (**b**) mean contraction amplitude (MCA) and spontaneous contraction variability (SCV), represented as error bars in the MCA plot and contraction percentage variation for the control (BSMA) for each considered condition; not significant differences (*p* > 0.05) between MCAs at different concentrations are reported in the graph. All the unreported comparisons are considered significant (*p* < 0.05).

**Figure 11:**
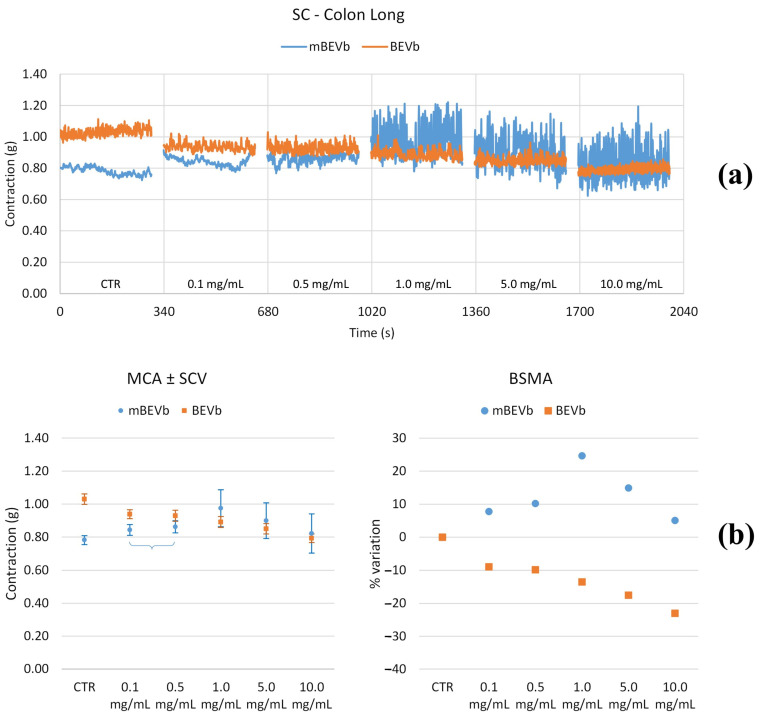
Experimental original recording of the concentration–response curve of BEVb and mBEVb on spontaneous longitudinal (Long) colon basal contractility. (**a**) Spontaneous contraction (SC) signals for each concentration; (**b**) mean contraction amplitude (MCA) and spontaneous contraction variability (SCV), represented as error bars in the MCA plot and contraction percentage variation for the control (BSMA) for each considered condition; not significant differences (*p* > 0.05) between MCAs at different concentrations are reported in the graph. All the unreported comparisons are considered significant (*p* < 0.05).

**Figure 12:**
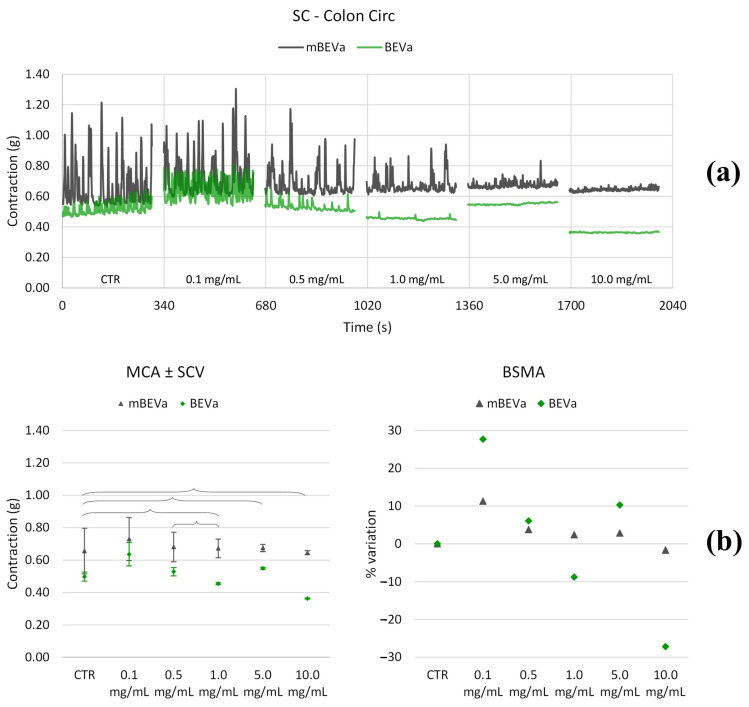
Experimental original recording of the concentration–response curve of BEVa and mBEVa on spontaneous circular (Circ) colon basal contractility. (**a**) Spontaneous contraction (SC) signals for each concentration; (**b**) mean contraction amplitude (MCA) and spontaneous contraction variability (SCV), represented as error bars in the MCA plot and contraction percentage variation for the control (BSMA) for each considered condition; not significant differences (*p* > 0.05) between MCAs at different concentrations are reported in the graph. All the comparisons not reported are to be considered significant (*p* < 0.05).

**Figure 13:**
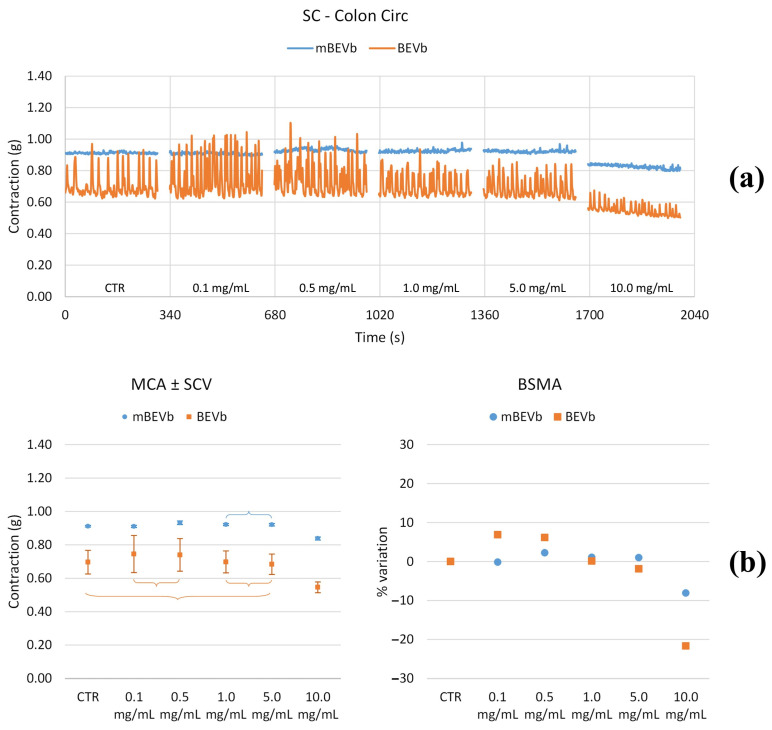
Experimental original recording of the concentration–response curve of BEVb and mBEVb on spontaneous circular (Circ) colon basal contractility. (**a**) Spontaneous contraction (SC) signals for each concentration; (**b**) mean contraction amplitude (MCA) and spontaneous contraction variability (SCV), represented as error bars in the MCA plot and contraction percentage variation for the control (BSMA) for each considered condition; not significant differences (*p* > 0.05) between MCAs at different concentrations are reported in the graph. All the comparisons not reported are to be considered significant (*p* < 0.05).

As depicted in Figure 10, Figure 11, Figure 12 and Figure 13 and summarized in Table 4 and Table 5, the longitudinal tone decreased at all concentrations of BEVa, and the circular tone decreased only at high concentrations (10.0 mg/mL); transit speed, pain, mixing, and fragmentation decreased starting from 0.5 mg/mL concentration. A different behavior was observed for the corresponding macerate (mBEVa) since the longitudinal tone decreased only at low concentrations (0.5 mg/mL), and the circular tone increased slightly at low concentrations, then remaining unchanged; transit speed and pain increased starting from 0.5 mg/mL while mixing and fragmentation decreased. When the muscle was treated with BEVb, the longitudinal tone decreased; the circular tone decreased only at high concentrations (10.0 mg/mL); transit speed increased or remained constant up to 5.0 mg/mL and then decreased at 10.0 mg/mL; pain decreased at low concentrations and then remained unchanged; mixing and fragmentation increased at low concentrations and decreased at high concentrations (10.0 mg/mL). Finally, the longitudinal tone increased at low mBEVb concentrations, while the circular tone increased only for intermediate concentrations; transit speed, pain, mixing, and fragmentation increased at all concentrations.

### 3.4. Metabolites Profiling of Lentil Hulls

In the present study, the metabolic profile of the most active lentil hull extract (BEVa) was studied, with the final aim of identifying the secondary metabolites possibly responsible for the observed biological activities.

The samples were lyophilized and resuspended in an H_2_O:MeOH mixture for liquid chromatography/tandem mass spectrometry (LC-MS/MS) analysis. The MS spectra were then processed using the commercial software Compound Discoverer v. 3.3.1.111 SP1 (Thermo Fisher, Bremen, Germany), and molecule identification was accomplished by screening ChemSpider and mzCloud. The ChemSpider node was used to identify putative compounds at a medium stringency level based on the accurate mass of the precursor ion, while for better confidence in identification, the mzCloud node was used based on fragment recognition of the most intense ions in the mass spectrum.

Owing to the intrinsic characteristics of the two identification tools applied, it was possible to identify a multitude of compounds according to the “level IIa” (probable structure = more advanced) and “level III” (putatively characterized = medium) criteria established by the Metabolomic Standard Initiatives [32,33]. Each molecule was searched in the PubChem online database for collecting information about its class and function.

Compound Discoverer totally identified 1292 compounds in Eston Green lentil hull extract. After a careful visual analysis of the spectra of the identified compounds, only 208 were internally validated and attributed to the extract under analysis. Specifically, 126 compounds were selected from ChemSpider results, while 159 molecules were obtained from mzCloud search. Each of these molecules was biologically relevant based on information available in the literature. In Table 6, the most relevant biological activities identified by ChemSpider and mzCloud searches of lentil hulls are listed, confirming the presence of numerous beneficial compounds such as alkaloids, aminoacids, peptides, phenolic, and others.

**Figure 14:**
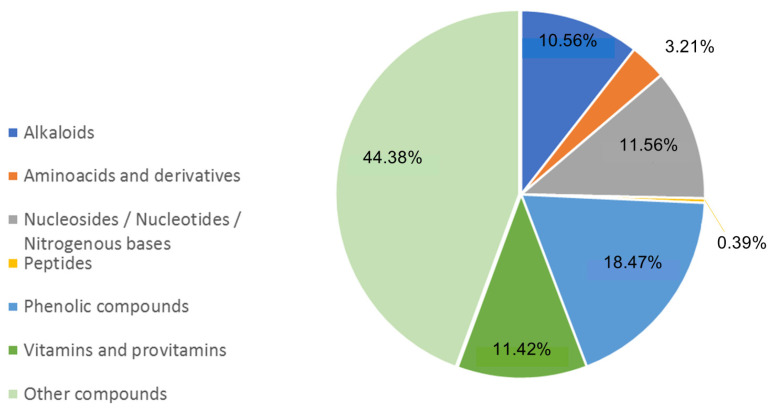
Relative abundance percentage of the most relevant classes of compounds that were identified in Eston Green lentil hulls according to the results obtained using Compound Discoverer software.

Furthermore, data retrieved from Compound Discoverer v. 3.3.1.111 SP1 (Thermo Fisher, Bremen, Germany) were used to perform a semi-quantitative analysis of the identified classes using the peak areas of each identified compound and screening the Chemspider and mzcloud DBs [27]. In Figure 14, the percentage distribution of the major classes of compounds identified and likely endowed with biological activity is pictured.

As can be seen in Figure 14, the most representative class of compounds endowed with biological properties found in BEVa is phenolic compounds (18.5%). Next, the classes of alkaloids, vitamins and provitamins, nucleotides/nucleosides and nitrogenous bases showed a similar relative abundance of approximately 11%. Finally, the class of amino acids and derivatives represented 3.21% of all the identified molecules.

## 4. Discussion

In the last few decades, there has been a growing interest in agro-industrial waste valorization, a process aimed at converting waste generated from agro-industrial activities into high-value products [34]. Hence, environmental impact is minimized and new economic opportunities are created by transforming waste into useful materials. Since agro-industrial waste often contains bioactive compounds with potential health benefits, solvent extraction processes allow their recovery and subsequent reuse in the pharmaceutical and nutraceutical fields, thus promoting the circular economy [35].

Recently, we proposed lentil hulls, usually the discarded outermost part of the legume seeds obtained from the decortication process, as a source of antioxidant phytochemicals [27]. Since the anti-inflammatory properties of lentil hull extracts have already been reported in the literature [17,20], we were interested in evaluating their possible application in the treatment of IBD for which a spasmolytic effect could be useful as well.

Based on our previous experience with microwave-assisted extraction (MAE) of bioactive compounds from different plant matrices [27,36,37,38,39], Eston green lentil hulls underwent extraction under microwave irradiation, and maceration was chosen as a comparative technique. As regards MAE, the main advantages of this technique are high extraction yield, high efficiency, and remarkable reduction of extraction time and solvent consumption [40,41]. To recover the widest possible composition of secondary metabolites, besides those endowed with antioxidant activity and usually extracted with water, three solvents with different polarity degrees were selected: water, abs EtOH, and ethyl acetate (EtOAc). Notably, a green extraction procedure was planned, with both an innovative green procedure (MAE) [42] and green solvents [43] having been identified. Unfortunately, lentil hulls were not manageable in water at the concentrations reached, since an excessive gelling of the suspension—conceivably caused by the high fiber content—was observed. Therefore, only abs EtOH and EtOAc were used in both extraction techniques (Table 1). A comparable extraction yield was reached in the four extraction procedures.

Knowing the anti-inflammatory potential of phenolic compounds, the total phenolic content of the four obtained extracts was evaluated as a preliminary investigation step through the well-known Folin–Ciocalteu assay. As reported in Table 1, and as could be expected based on the hydrophilic nature of polyphenols, a higher total phenolic content was observed when abs EtOH was used, regardless of the extraction process applied. On the other hand, a higher content of polyphenols was reached working under microwave irradiation in comparison with maceration, as already reported in our previous works [27,36,37,38,39], with the greater efficiency of MAE being one of the well-known main advantages of this technique. The very close TPC values registered with EtOAc (entry 2 vs. 4) could be due to its low affinity toward the more hydrophilic phenols, regardless of the extraction process used.

The intestinal cells are able to elicit inflammatory responses, releasing a number of mediators, to protect the host against potentially dangerous infections [44]. In this regard, the intestinal mucosa exhibits a low-grade, almost physiologic inflammation due to a great continuous antigenic exposure to luminal bacteria as well as the expression of a variety of TLR receptors, including TLR4 that specifically binds to LPS [45]. In the present work, we observed a significant increase in TLR4 after endotoxin treatment of Caco-2 cells, thus demonstrating that the responsiveness to LPS of the intestinal epithelial cells is positively correlated with TLR4 expression, as previously described [46,47]. Interestingly, in our experimental model, all extracts tested were able to down-regulate the TLR4 expression in LPS-stimulated cells with the extracts obtained under microwave irradiation showing, at all tested doses, a more significant ability to weaken the receptor expression and, therefore, modulate cell inflammatory responses.

It is well known that excessive inflammatory responses may alter the intestinal mucosa homeostasis destroying the bowel epithelial monolayer. Moreover, inflammatory diseases are often associated with the overexpression of NO and pro-inflammatory cytokines [48]. NO is responsible for various cellular functions of the gastrointestinal mucosa, including maintenance of perfusion, regulation of microvascular and epithelial permeability, and immune functions [49]. Although controlled NO production by intestinal cells contributes to the normal homeostasis of the intestinal epithelium, excessive production of NO due to iNOS can cause damage to the intestinal mucosa [50]. The enzyme nitric oxide synthase is responsible for the increased production of NO from L-arginine and causes an inflammatory state in the intestinal epithelium, causing protein nitrosylation and nitrative damage to DNA, resulting in the death of intestinal epithelial cells [50]. In our experimental model, we observed an increase in iNOS expression in LPS-activated cells that were significantly down-regulated by the pre-treatment of Caco-2 cells with all lentil hull extracts, with the microwave extracts showing a more effective ability in reducing this expression. Thus, a reduction in iNOS expression could play a key role in the anti-inflammatory properties of lentil hull extracts.

An increasing amount of evidence demonstrates that IBDs are often associated with the excessive production of inflammatory cytokines, such as IL-1β that contribute to impair intestinal permeability [51,52]. This effect may probably be due to potential mechanisms exerted by pro-inflammatory cytokines leading to a disruption of the intestinal barrier, such as a reduced expression of tight-junctions-associated proteins [53,54]. Being generally known that LPS can stimulate the pro-inflammatory cytokines, it is often used to set up an inflammation-associated model. Therefore, in our in vitro model, we observed that Caco-2 cells exposed to LPS treatment underwent a significant overproduction of IL-1 that was negatively modulated after treatment with all hull extracts tested. Also in this case, we observed that MAE seems to be the most effective in reducing IL-1 production in LPS-treated Caco-2 cells.

Finally, IL-10 is an important anti-inflammatory cytokine that has been demonstrated to be widely involved in IBDs. In fact, it was reported that the expression of IL-10 in IBD patients is lower in comparison to normal people [55]. In this respect, this anti-inflammatory cytokine is able to inhibit macrophage activation containing inflammatory response [55,56]. Interestingly, our results demonstrated that LPS-activated cells submitted to pre-treatment with lentil hull extracts exhibited increased levels of the anti-inflammatory cytokine IL-10. Moreover, among all extracts tested, those obtained through MAE were, once again, more effective to upregulate the IL-10 production in the treated intestinal cells. Therefore, the lentil hull extracts, especially those obtained under microwave irradiation, seem to exhibit potential protective effects towards intestinal epithelial cells, down-regulating pro-inflammatory mediators and, at the same time, upregulating anti-inflammatory responses.

Knowing that IBDs are characterized by inflammatory lesions mainly affecting the ileum and colon, with the pharmacotherapy being based on the combination of anti-inflammatory drugs with contractility correctors [57], a possible lentil hull extract spasmolytic effect has also been investigated. Based on our previous experience [57], low frequencies (LF) related to longitudinal smooth muscle are linked to transit speed, while mid frequencies (MF) and high frequencies (HF) are likely associated with pain. Regarding circular smooth muscle, LF contributes to mixing, while MF and HF could be related to the fragmentation capacity of the food bolus. Concerning the ileum, our findings demonstrate that lentil hull extracts have differentiated effects on longitudinal and circular muscles. For instance, the mBEVb extract increases longitudinal tone at low concentrations, while circular tone significantly decreases at all concentrations. In contrast, the BEVa extract elicits a significant decrease in longitudinal and circular tones at low concentrations. These results suggest that lentil hull extracts may influence intestinal motility depending on concentration and extract type. Moreover, we observed variations in parameters associated with intestinal function, such as transit speed, pain, mixing capacity, and bolus fragmentation. For example, the BEVa extract showed beneficial effects, reducing transit speed and pain at the ileum level and decreasing transit speed, pain, and muscular tone at the colon level, even at low concentrations. Regarding the colon, our results indicate that lentil hull extracts may affect muscle tone and function similarly to the ileum but with some significant differences. For instance, the mBEVb extract increased longitudinal tone at low concentrations and circular tone only at intermediate concentrations. Conversely, the BEVb extract exhibited a more complex action, with decreased longitudinal and circular tone and variations in transit speed, pain, and the capacity to mix and fragment the food bolus at different concentrations.

Overall, biological results highlighted the potential of BEVa as a promising adjunctive therapy for managing IBDs, thus making a metabolic characterization of this extract needful to identify the compounds conceivably accounting for the observed activities.

As reported in Table 6 and Figure 14, BEVa contains many compounds with high nutritional value among which are phenolic compounds, alkaloids, vitamins and provitamins, nucleotides/nucleosides, and nitrogenous bases. Conversely, few peptides were identified in the extract, with this result possibly stemming from the lipophilic nature of the extraction solvent used (EtOAc), as opposed to what was observed for the aqueous lentil hull extract investigated in our previous work [27].

It is worth noting that, although the relative abundance of the alkaloid class was very high, only two compounds were identified, namely, sinapine and tetramethylpyrazine. Therefore, further investigations are required to clarify whether MAE extraction with ethyl acetate improves the extraction of these compounds or whether they are actually more abundant than the other molecules identified in the Eston green lentils. In particular, sinapine, found in different plant species, is an alkaloid endowed with anti-inflammatory and antioxidant properties, apart from neuroprotective and anticancer properties [58].

**Figure 15:**
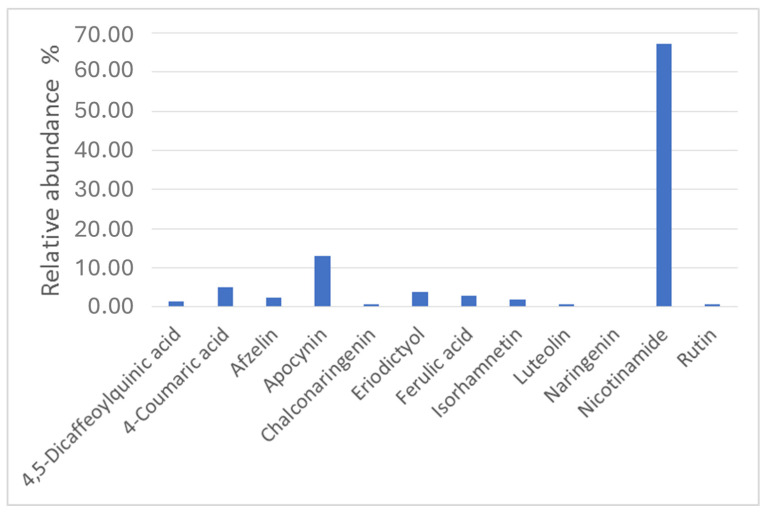
Relative abundance percentage of anti-inflammatory compounds identified in Eston Green lentil hulls based on results obtained using Compound Discoverer software.

A thorough literature search allowed us to identify twelve compounds, among those identified by LC-HR MS/MS, as notoriously endowed with anti-inflammatory properties, with a total abundance of approximately 7.35%. The relative abundance is depicted in Figure 15.

Although most of the identified anti-inflammatory compounds belong to the class of polyphenols, both the water-soluble vitamin nicotinamide and the structurally vanillin-linked apocyanin were the most abundant detected, with a relative abundance of 67.4% and 13%, respectively. 4-Coumaric acid, a natural hydroxycinnamic acid, showed an abundance of approximately 5%. 4,5-Dicaffeoylquinic acid, afzelin, chalconaringenin, eriodictyol, ferulic acid, isorhamnetin, luteolin, naringenin, and rutin showed lower abundances (less than 5%).

The three most abundant compounds have been previously associated with anti-inflammatory and smooth muscle-modulating properties. Niacinamide (or nicotinamide), also known as vitamin B3, having the highest content in the extract, has been shown to possess anti-inflammatory effects by inhibiting pro-inflammatory cytokines and reducing oxidative stress [59,60]. It also induced relaxing effects on the smooth muscle of the ileum patients, resulting in both pain and inflammation reduction [61,62,63,64]. Apocynin (or acetovanillone), traditionally used in Ayurvedic medicine [65], is a natural compound with demonstrated antioxidant and anti-inflammatory properties stemming from the inhibition of the production of reactive oxygen species (ROS) and inflammatory mediators [66,67]. In particular, apocynin is known for inhibiting the enzyme NOS (nitric oxide synthase), responsible for nitric oxide (NO) production, with NO being involved in the regulation of intestinal inflammation and motility [68]. Actually, apocynin’s therapeutic role has been demonstrated in chronic inflammatory bowel diseases such as Crohn’s disease and ulcerative colitis [69]. 4-Coumaric acid is a phenolic compound also endowed with anti-inflammatory and antioxidant properties [70,71,72,73] resulting from the reduction of the expression of inflammatory mediator factors [70]. In particular, the protection effect against intestinal inflammation in rats [74] and the antispasmodic action [75] have been described. Despite the moderate content of each of the above three compounds (≤5%), a possible synergistic effect may be anticipated since, generally, complex mixtures of herbal secondary metabolites display higher activity than the corresponding isolated compounds [76]. Overall, the synergistic action of niacinamide, apocynin, and *p*-coumaric acid in BEVa underscores its potential as a therapeutic agent for IBDs. Further research is warranted to elucidate the mechanisms underlying the beneficial effects of BEVa and its constituent molecules in managing IBDs.

## 5. Conclusions

Food wastes or by-products are attracting increasing attention as a source of functional food ingredients for human consumption [77]. Hulls, pods, and husks from various sources (including cocoa, pea, peanut, sunflower, chickpea, rice, soybean, sesame, bean, carob, oat, and coffee) have been studied in this respect [78,79]. Our study suggests that lentil hull may be relevant to functional foods whose consumption could contribute to overall intestinal health maintenance. Our results point out that MAE allows for the efficient extraction of biologically active compounds without significant degradation when working at high temperatures but for short times, with this technique proving more efficient than the traditional one (maceration). Anti-inflammatory and spasmolytic activities were observed for all the obtained extracts with BEVa conceivably being the most potent in managing the IBD symptoms. It is noteworthy that more than 7% of all the metabolites identified by the bioinformatic tools were attributed to compounds with proven anti-inflammatory and antispasmodic activities, with niacinamide, apocynin, and 4-coumaric acid being the most abundant. On the other hand, mBEVb is also noteworthy, being responsible for the increase in transit speed, mixing, and fragmentation at all concentrations, together with the increase in both longitudinal and circular colon tone, thus proving possibly useful for irritable bowel syndrome with constipation (IBS-C) treatment. All in all, our findings provide a compelling basis for further exploring lentil hull extracts’ potential in intestinal barrier dysfunction management, paving the way for developing new integrated therapeutic strategies in a future perspective.

## Figures and Tables

**Table 1:** Experimental extraction conditions and total phenolic content of Eston green lentil hull extracts.

Entry	Method	Solvent	Extract Abbreviation	Yield (%) ^a^	TPC ^b^
1	MAE	abs EtOH	BEVb	4.5	2.14 ± 0.02
2	MAE	EtOAc	BEVa	4.1	0.22 ± 0.05
3	maceration	abs EtOH	mBEVb	4.6	1.30 ± 0.08
4	maceration	EtOAc	mBEVa	4.3	0.35 ± 0.07

^a^ Yield% = (weight of the dry extract/weight of the dry plant) × 100; ^b^ Total phenolic content expressed as mg GAE/g dry weight. Values represent means ± SD (*n* = 3).

**Table 2:** Results of frequency domain investigation for ileum. (*: no % difference).

		Concentration (mg/mL)	BEVa	mBEVa	BEVb	mBEVb
Ileum long	LF	0.1	*	*	*	−70%
		0.5	*	−90%	*	−90%
		1.0	−70%	−70%	*	*
		5.0	−90%	−60%	*	*
		10.0	−100%	*	*	*
	MF	0.1	*	*	*	*
		0.5	−60%	−70%	*	*
		1.0	−80%	−70%	*	*
		5.0	−100%	−70%	*	+100%
		10.0	−100%	−60%	*	*
	HF	0.1	*	*	*	+100%
		0.5	−50%	*	*	*
		1.0	−50%	*	*	+70%
		5.0	−70%	*	*	*
		10.0	−100%	*	*	−60%
Ileum circ	LF	0.1	>+500%	*	*	−100%
		0.5	>+500%	+100%	*	−100%
		1.0	>+500%	*	*	−100%
		5.0	>+500%	+50%	*	−100%
		10.0	*	*	>+500%	−100%
	MF	0.1	+60%	*	−100%	*
		0.5	+290%	*	*	+80%
		1.0	+130%	*	*	*
		5.0	−60%	*	*	+70%
		10.0	−100%	*	+70%	+290%
	HF	0.1	+100%	*	*	*
		0.5	+150%	*	*	*
		1.0	+140%	*	*	*
		5.0	−60%	*	*	*
		10.0	−90%	*	+60%	+100%

LF (low frequency), MF (mid frequency), and HF (high frequency) percentage variations were classified depending on their value following these criteria: ≤−500%: “---”; [−500%,−300%]: “--”; [−300%,−50%]: “-“; [−50%,+50%]: “=”; [+50%,+300%]: “+”; [+300%,+500%]: “++”; ≥+500%: “+++”. Similarly, tone variations were categorized as follows: ≤−100%: “------”; [−100%,−80%]: “-----”; [−80%,−60%]: “----”; [−60%,−40%]: “---”; [-40%,−20%]: “--”; [−20%,0%]: “-“; Not significant: “=”; [0%,+20%]: “+”; [+20%,+40%]: “++”; [+40%,+60%]: “+++”; [+60%,+80%]: “++++”; [+80%,+100%]: “+++++”; ≥+100%: “++++++”.

**Table 3:** Effects on the ileum of the different compounds on transit speed, pain, mixing, fragmentation, and tones.

ILEUM		Transit Speed Variation %	Pain %	Mixing %	Fragmentation %	Longitudinal Contraction Variation %	Circular Contraction Variation %
BEVa	0.1 mg/mL	=	=	+++	+	-	-
	0.5 mg/mL	=	-	+++	+	-	-
	1.0 mg/mL	-	-	+++	+	-	-
	5.0 mg/mL	-	-	+++	-	-	-
	10.0 mg/mL	-	-	=	-	--	-
mBEVa	0.1 mg/mL	=	=	=	=	-	-
	0.5 mg/mL	-	-	+	=	-	-
	1.0 mg/mL	-	-	=	=	-	-
	5.0 mg/mL	-	-	+	=	-	-
	10.0 mg/mL	=	-	=	=	=	-
BEVb	0.1 mg/mL	=	=	=	-	-	-
	0.5 mg/mL	=	=	=	=	-	--
	1.0 mg/mL	=	=	=	=	-	--
	5.0 mg/mL	=	=	=	=	-	--
	10.0 mg/mL	=	=	+++	+	-	--
mBEVb	0.1 mg/mL	-	+	-	=	=	--
	0.5 mg/mL	-	=	-	+	+	---
	1.0 mg/mL	=	+	-	=	+	---
	5.0 mg/mL	=	+	-	+	+	----
	10.0 mg/mL	=	-	-	+	++	----

LF (low frequency), MF (mid frequency), and HF (high frequency) percentage variations were classified depending on their value following these criteria: ≤−500%: “---”; [−500%,−300%]: “--”; [−300%,−50%]: “-“; [−50%,+50%]: “=”; [+50%,+300%]: “+”; [+300%,+500%]: “++”; ≥+500%: “+++”. Similarly, tone variations were categorized as follows: ≤−100%: “------”; [−100%,−80%]: “-----”; [−80%,−60%]: “----”; [−60%,−40%]: “---”; [-40%,−20%]: “--”; [−20%,0%]: “-“; Not significant: “=”; [0%,+20%]: “+”; [+20%,+40%]: “++”; [+40%,+60%]: “+++”; [+60%,+80%]: “++++”; [+80%,+100%]: “+++++”; ≥+100%: “++++++”.

**Table 4:** Results of frequency domain investigation for Colon. (*: no % difference).

		Concentration (mg/mL)	Concentration (mg/mL)	BEVa	mBEVa	BEVb	mBEVb
Colon long	LF	0.1	*	*	+100%	*	+100%
		0.5	+250%	+250%	−60%	>+500%	*
		1.0	>+500%	>+500%	−80%	>+500%	+120%
		5.0	>+500%	>+500%	−90%	>+500%	*
		10.0	>+500%	>+500%	−90%	>+500%	−50%
	MF	0.1	*	*	*	*	−50%
		0.5	>+500%	>+500%	−80%	+420%	*
		1.0	>+500%	>+500%	−100%	>+500%	*
		5.0	>+500%	>+500%	−100%	>+500%	*
		10.0	>+500%	>+500%	−100%	>+500%	*
	HF	0.1	*	*	*	*	*
		0.5	+165%	+165%	−80%	*	*
		1.0	+350%	+350%	−90%	+190%	*
		5.0	+480%	+480%	−100%	+400%	*
		10.0	+400%	+400%	−90%	>+500%	*
Colon circ	LF	0.1	0.1	>+500%	*	+150%	*
		0.5	0.5	*	−70%	+100%	+80%
		1.0	1.0	−90%	−90%	*	+120%
		5.0	5.0	−100%	−100%	*	*
		10.0	10.0	−100%	−100%	−90%	+60%
	MF	0.1	0.1	>+500%	*	+250%	+210%
		0.5	0.5	−70%	*	+230%	+210%
		1.0	1.0	−100%	−50%	+90%	+70%
		5.0	5.0	−100%	−90%	*	+230%
		10.0	10.0	−100%	−100%	−70%	+200%
	HF	0.1	0.1	+500%	*	>+500%	+70%
		0.5	0.5	−60%	−60%	+240%	+80%
		1.0	1.0	−90%	−90%	+180%	+60%
		5.0	5.0	−90%	−100%	+130%	+70%
		10.0	10.0	−90%	−100%	*	+80%

**Table 5:** Effects on the colon of the different compounds include transit speed, pain, mixing, fragmentation, and muscle tones.

COLON		Transit Speed Variation %	Pain %	Mixing %	Fragmentation %	Longitudinal Contraction Variation %	Circular Contraction Variation %
BEVa	0.1 mg/mL	+	=	+++	+++	-	++
	0.5 mg/mL	-	-	=	-	--	+
	1.0 mg/mL	-	-	-	-	--	-
	5.0 mg/mL	-	-	-	-	--	+
	10.0 mg/mL	-	-	-	-	--	--
mBEVa	0.1 mg/mL	=	=	=	=	+	+
	0.5 mg/mL	+++	++	-	-	--	+
	1.0 mg/mL	+++	+++	-	-	-	=
	5.0 mg/mL	+++	+++	-	-	-	=
	10.0 mg/mL	+++	+++	-	-	-	=
BEVb	0.1 mg/mL	+	-	+	+++	-	+
	0.5 mg/mL	=	=	+	+	-	+
	1.0 mg/mL	+	=	=	+	-	=
	5.0 mg/mL	=	=	=	+	-	=
	10.0 mg/mL	-	=	-	-	--	--
mBEVb	0.1 mg/mL	=	=	=	+	+	=
	0.5 mg/mL	+	+++	+	+	+	+
	1.0 mg/mL	+++	+++	+	+	++	+
	5.0 mg/mL	+++	+++	=	+	+	+
	10.0 mg/mL	+++	+++	+	+	+	-

LF (low frequency), MF (mid frequency), and HF (high frequency) percentage variations were classified depending on their value following these criteria: ≤−500%: “---”; [−500%,−300%]: “--”; [−300%,−50%]: “-“; [−50%,+50%]: “=”; [+50%,+300%]: “+”; [+300%,+500%]: “++”; ≥+500%: “+++”. Similarly, tone variations were categorized as follows: ≤−100%: “------”; [−100%,−80%]: “-----”; [−80%,−60%]: “----”; [−60%,−40%]: “---”; [-40%,−20%]: “--”; [−20%,0%]: “-“; Not significant: “=”; [0%,+20%]: “+”; [+20%,+40%]: “++”; [+40%,+60%]: “+++”; [+60%,+80%]: “++++”; [+80%,+100%]: “+++++”; ≥+100%: “++++++”.

**Table 6:** Summary of the most relevant molecules identified in the hulls of Eston green lentils after processing high-resolution mass spectrometry (HRMS) spectra using Compound Discoverer software. The level of identification medium * (level III—putatively characterized) and advanced ** (level IIa—probable structure) were specified for each molecule along with the biological class they belong to.

Class	Name	Chemical Formula	Adduct	*m*/*z*	RT (min)	Identification Level
Alkaloids	Sinapine	C_16_H_23_NO_5_	[M + H]^+^	310.16486	32.376	**
	Tetramethylpyrazine	C_8_H_12_N_2_	[M + H]^+^	137.1073	46.486	**
Aminoacids and derivatives	Gamma-hydroxyhomoarginine	C_7_H_16_N_4_O_3_	[M + H]^+^	205.12943	1.871	*
	Histidinate	C_6_H_8_N_3_O_2_	[M − H]^−^	153.05456	14.859	*
	l-Tyrosine methyl ester	C_10_H_13_NO_3_	[M − H]^−^	194.08155	19.552	**
	*N*-Acetyl-l-phenylalanine	C_11_H_13_NO_3_	[M − H]^−^	206.08167	45.068	**
Nucleosides/Nucleotides/Nitrogenous bases	Adenine	C_5_H_5_N_5_	[M + H]^+^	136.06175	7.539	**
	Adenosine	C_10_H_13_N_5_O_4_	[M + H]^+^	268.1039	7.543	**
	Thymidine	C_10_H_14_N_2_O_5_	[M − H]^−^	241.08281	12.52	*
	Uracil	C_4_ H_4_N_2_O_2_	[M + H]^+^	113.03482	5.256	**
	Uric acid	C_5_H_4_N_4_O_3_	[M + H]^+^	169.03555	4.157	**
	Uridine	C_9_H_12_N_2_O_6_	[M − H]^−^	243.06219	5.248	**
Peptides	Beta-Ala-Lys-*N*(epsilon)-AMCA	C_21_H_28_N_4_O_6_	[M − H]^−^	431.19264	46.18	*
	Trp-asp-glu	C_20_H_24_N_4_O_8_	[M − H]^−^	447.15133	40.191	*
Phenolic compounds	2-[({[4-({[(2-Hydroxyphenyl)methylene]amino}methyl)cyclohexyl]methyl}imino)methyl]phenol	C_22_H_26_N_2_O_2_	[M + H]^+^	351.20609	49.224	**
	3,4-Dihydroxybenzaldehyde	C_7_H_6_O_3_	[M + H]^+^	139.03893	20.307	**
	3,5-Dihydroxy-2-(4-hydroxyphenyl)-4-oxo-3,4-dihydro-2*H*-chromen-7-yl hexopyranoside	C_21_H_22_O_11_	[M − H]^−^	449.10943	34.313	**
	4-(Isothiocyanatomethyl)phenol	C_8_H_7_NOS	[M + H]^+^	166.0321	47.176	*
	4-(Phenylethynyl)phenol	C_14_H_10_O	[M + H]^+^	195.08044	53.625	*
	4,5-Dicaffeoylquinic acid	C_25_H_24_O_12_	[M − H]^−^	515.11992	51.061	**
	4-Coumaric acid	C_9_H_8_O_3_	[M + H]^+^	165.05458	30.554	**
	6-*O*-[(2*E*)-3-(4-Hydroxyphenyl)-2-propenoyl]-1-*O*-(3,4,5-trihydroxybenzoyl)hexopyranose	C_22_H_22_O_12_	[M − H]^−^	477.10409	53.366	**
	Afzelin	C_21_H_20_O_10_	[M − H]^−^	431.09862	53.643	**
	Apigenin 7-(3”-acetyl-6”-E-p-coumaroylglucoside)	C_32_H_28_O_13_	[M + H]^+^	621.15947	45.897	*
	Apocynin	C_9_H_10_O_3_	[M + H]^+^	167.07021	44.149	**
	Astragalin	C_21_H_20_O_11_	[M − H]^−^	447.09416	53.331	**
	Catechin	C_15_H_14_O_6_	[M − H]^−^	289.07211	27.963	**
	Chalconaringenin	C_15_H_12_O_5_	[M + H]^+^	273.0754	49.069	*
	Eriodictyol	C_15_H_12_O_6_	[M + H]^+^	289.07028	53.188	**
	Ferulic acid	C_10_H_10_O_4_	[M − H]^−^	193.04989	47.919	**
	Hyperoside	C_21_H_20_O_12_	[M + H]^+^	465.10236	52.356	**
	Isorhamnetin	C_16_H_12_O_7_	[M + H]^+^	317.06503	53.379	**
	Kaempferol	C_15_H_10_O_6_	[M + H]^+^	287.0547	53.638	**
	Luteolin	C_15_H_10_O_6_	[M − H]^−^	285.04052	53.883	**
	Mitoxantrone	C_22_H_28_N_4_O_6_	[M − H]^−^	443.19273	29.803	*
	Myricetin 3-*O*-beta-d-galactopyranoside	C_21_H_20_O_13_	[M − H]^−^	479.08365	49.938	**
	Myricitrin	C_21_H_20_O_12_	[M − H]^−^	463.08897	51.464	**
	Naringenin	C_15_H_12_O_5_	[M − H]^−^	271.06119	53.81	**
	Naringeninchalcone	C_15_H_12_O_5_	[M + H]^+^	273.07531	51.595	**
	*N*-Feruloyloctopamine	C_18_H_19_NO_5_	[M − H]^−^	328.11941	52.055	*
	Phaseolic acid	C_12_H_22_O_6_	[M − H]^−^	261.13446	51.523	*
	Phloretin	C_15_H_14_O_5_	[M + H]^+^	275.09114	53.461	*
	Quercetin	C_15_H_10_O_7_	[M + H]^+^	303.04963	53.338	**
	Quercetin-3β-d-glucoside	C_21_H_20_O_12_	[M − H]^−^	463.08923	52.362	**
	Resveratrol	C_14_H_12_O_3_	[M + H]^+^	229.08556	48.286	**
	Rutin	C_27_H_30_O_16_	[M − H]^−^	609.14705	50.539	**
	Syringaldehyde	C_9_H_10_O_4_	[M + H]^+^	183.06521	41.581	*
	Taxifolin	C_15_H_12_O_7_	[M + H]^+^	305.06522	51.303	**
Vitamins and provitamins	Choline	C_5_H_13_NO	[M + H]^+^	104.10739	1.877	**
	Nicotinamide	C_6_H_6_N_2_O	[M + H]^+^	123.05548	3.465	**
	Pantothenic acid	C_9_H_17_NO_5_	[M − H]^−^	218.10293	14.431	**
Other compounds	(±)-Abscisic acid	C_15_H_20_O_4_	[M − H]^−^	263.12911	53.545	**
	1-(4-Methylphenyl)pyrrolidine-2,5-dione	C_11_H_11_NO_2_	[M + H]^+^	190.08625	53.563	**
	1-(Carboxymethyl)cyclohexanecarboxylic acid	C_9_H_14_O_4_	[M + H]^+^	187.09649	51.399	**
	1*H*-Pyrazole-4-carbonitrile	C_4_H_3_N_3_	[M − H]^−^	92.0252	49.38	*
	1-Methyl-4-(1-methyl-2-propenyl)-benzene	C_13_H_18_	[M + H]^+^	175.14814	53.606	*
	2-(1*H*-Indol-3-yl)acetic acid	C_10_H_9_NO_2_	[M + H]^+^	176.07055	52.517	**
	2,4-Heptadienal	C_7_H_10_O	[M + H]^+^	111.08073	52.938	*
	2,5,8,11,14,17-Hexaoxaoctadecane	C_12_H_26_O_6_	[M + Na]^+^	289.16181	44.217	*
	2,6-Dimethylpyrazine	C_6_H_8_N_2_	[M + H]^+^	109.07632	2.042	**
	2-Hydroxydecanedioic acid	C_10_H_18_O_5_	[M − H]^−^	217.10763	52.081	*
	3-(2-Hydroxyethyl)indole	C_10_H_11_NO	[M + H]^+^	162.09119	49.171	**
	4-Dimethylaminocinnamaldehyde	C_11_H_13_NO	[M + H]^+1^	176.1069	52.277	**
	4-Indolecarbaldehyde	C_9_H_7_NO	[M + H]^+^	146.05999	47.077	**
	4-Methoxycinnamaldehyde	C_10_H_10_O_2_	[M + H]^+^	163.07535	49.332	**
	4-Oxo-5-phenylpentanoic acid	C_11_H_12_O_3_	[M + H]^+^	193.08605	46.049	**
	4-Oxododecanedioic acid	C_12_H_20_O_5_	[M + H]^+^	245.13827	52.666	**
	5-Oxo-7-octenoic acid	C_8_H_12_O_3_	[M + H]^+^	157.08599	53.706	*
	8-Hydroxyquinoline	C_9_H_7_NO	[M + H]^+^	146.05998	23.334	**
	9-(Methylsulfinyl) nonanoic acid	C_10_H_20_O_3_S	[M − H]^−^	219.10549	53.471	*
	Asperitaconic acid C	C_11_H_16_O_5_	[M − H]^−^	227.09209	50.274	*
	Cyclo(phenylalanyl-prolyl)	C_14_H_16_N_2_O_2_	[M + H]^+^	245.12826	48.365	**
	d-(–)-Quinic acid	C_7_H_12_O_6_	[M − H − H_2_O]^−^	173.04454	2.096	**
	Dibenzylamine	C_14_H_15_N	[M + H]^+^	198.12771	46.296	**
	d-iditol	C_6_H_14_O_6_	[M + H]^+^	183.08638	1.945	*
	Dulcitol	C_6_H_14_O_6_	[M − H]^−^	181.07088	2.001	**
	Ethyl sorbate	C_8_H_12_O_2_	[M + H]^+^	141.09091	51.34	**
	Gabapentin	C_9_H_17_NO_2_	[M + H]^+^	172.1331	45.282	**
	Gluconic acid	C_6_H_12_O_7_	[M − H]^−^	195.05033	1.993	**
	Hmmtic	C_6_H_10_N_6_O_2_	[M + H]^+^	199.094	52.156	*
	Indole-3-lactic acid	C_11_H_11_NO_3_	[M − H]^−^	204.06601	46.717	**
	Lariciresinol 4-*O*-glucoside	C_26_H_34_O_11_	[M − H]^−^	521.2034	48.819	**
	Methyl 2*E*,4*E*,6*Z*-decatrienoate	C_11_H_16_O_2_	[M + H]^+^	181.12232	53.863	*
	Mexiletine	C_11_H_17_NO	[M + H]^+^	180.13823	48.866	**
	*N*-acetyldopamine	C_10_H_13_NO_3_	[M + H]^+^	196.09681	19.569	**
	*N*-acetyltyramine	C_10_H_13_NO_2_	[M + H]^+^	180.10183	27.328	**
	Nootkatone	C_15_H_22_O	[M + H]^+^	219.17417	53.659	**
	Oxepanone	C_6_H_10_O_2_	[M + H]^+^	115.0756	19.494	**
	Pimelic dialdehyde	C_7_H_12_O_2_	[M + H]^+^	129.09116	53.704	*
	Repirinast	C_20_H_21_NO_5_	[M − H]^−^	354.13494	1.078	*
	Sedanolide	C_12_H_18_O_2_	[M + H]^+^	195.13799	52.389	**
	Sjg 136	C_31_H_32_N_4_O_6_	[M − H]^−^	555.22424	52.552	*
	Sucrose	C_12_H_22_O_11_	[M − H]^−^	341.10925	2.072	**
	Tetraethylene glycol dimethyl ether	C_10_H_22_O_5_	[M+Na]^+^	245.13581	36.844	*
	*Trans*-Cinnamaldehyde	C_9_H_8_O	[M + H]^+^	133.06482	51.824	**
	Δ-Gluconic acid δ-lactone	C_6_H_10_O_6_	[M − H]^−^	177.03951	2.038	**

## Data Availability

The original contributions presented in the study are included in the article. Further inquiries can be directed to the corresponding author.
